# 1′-Methyl-4′-phenyldispiro­[indan-2,2′-pyrrolidine-3′,2′′-indan]-1,3,1′′-trione

**DOI:** 10.1107/S1600536811032934

**Published:** 2011-08-27

**Authors:** Ang Chee Wei, Mohamed Ashraf Ali, Tan Soo Choon, Ching Kheng Quah, Hoong-Kun Fun

**Affiliations:** a Institute for Research in Molecular Medicine, Universiti Sains Malaysia, 11800 USM, Penang, Malaysia; b X-ray Crystallography Unit, School of Physics, Universiti Sains Malaysia, 11800 USM, Penang, Malaysia

## Abstract

The conformation of the title compound, C_27_H_21_NO_3_, is stabilized by a weak intra­molecular C—H⋯O hydrogen bond, which generates an *S*(6) ring motif. The pyrrolidine ring adopts a half-chair conformation. Both of the other five-membered rings are in envelope conformations. No significant inter­molecular hydrogen bonds are observed.

## Related literature

For general background to and the biological activity of the title compound, see: Amalraj *et al.* (2003 ▶); Karthikeyan *et al.* (2010 ▶); Chande *et al.* (2005 ▶); Sriram *et al.* (2009 ▶); Duncan & Barry (2004 ▶). For reference bond-length data, see: Allen *et al.* (1987 ▶). For related structures, see: Kumar *et al.* (2010 ▶); Wei *et al.* (2011 ▶). For hydrogen-bond motifs, see: Bernstein *et al.* (1995 ▶). For ring conformations, see: Cremer & Pople (1975 ▶).
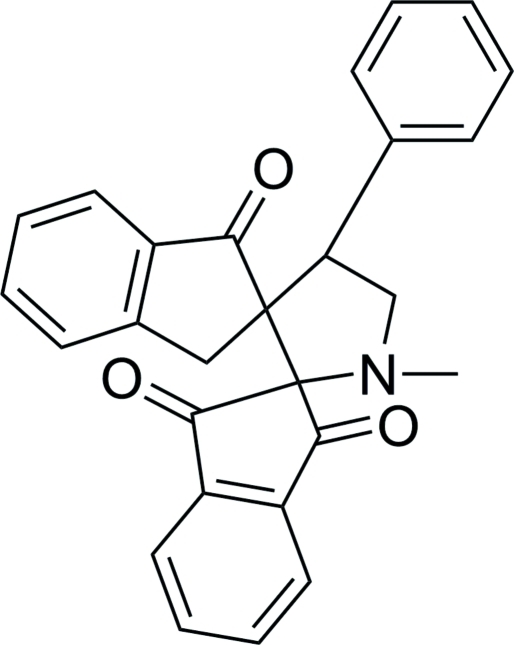


## Experimental

### 

#### Crystal data

C_27_H_21_NO_3_
*M* *_r_* = 407.45
Monoclinic, 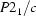

*a* = 8.4578 (7) Å
*b* = 11.6194 (9) Å
*c* = 22.6360 (16) Å
β = 109.693 (2)°
*V* = 2094.4 (3) Å^3^
*Z* = 4
Mo *K*α radiation
μ = 0.08 mm^−1^
*T* = 296 K
0.34 × 0.26 × 0.15 mm

#### Data collection

Bruker SMART APEXII DUO CCD area-detector diffractometer
Absorption correction: multi-scan (*SADABS*; Bruker, 2009 ▶) *T*_min_ = 0.972, *T*_max_ = 0.988
23854 measured reflections
6084 independent reflections
3982 reflections with *I* > 2σ(*I*)
*R*_int_ = 0.035

#### Refinement

*R*[*F* ^2^ > 2σ(*F* ^2^)] = 0.052
*wR*(*F* ^2^) = 0.163
*S* = 1.03
6084 reflections
281 parameters
H-atom parameters constrained
Δρ_max_ = 0.20 e Å^−3^
Δρ_min_ = −0.21 e Å^−3^

### 

Data collection: *APEX2* (Bruker, 2009 ▶); cell refinement: *SAINT* (Bruker, 2009 ▶); data reduction: *SAINT*; program(s) used to solve structure: *SHELXTL* (Sheldrick, 2008 ▶); program(s) used to refine structure: *SHELXTL*; molecular graphics: *SHELXTL*; software used to prepare material for publication: *SHELXTL* and *PLATON* (Spek, 2009 ▶).

## Supplementary Material

Crystal structure: contains datablock(s) global, I. DOI: 10.1107/S1600536811032934/wn2448sup1.cif

Structure factors: contains datablock(s) I. DOI: 10.1107/S1600536811032934/wn2448Isup2.hkl

Additional supplementary materials:  crystallographic information; 3D view; checkCIF report

## Figures and Tables

**Table 1:** Hydrogen-bond geometry (Å, °)

*D*—H⋯*A*	*D*—H	H⋯*A*	*D*⋯*A*	*D*—H⋯*A*
C27—H27*A*⋯O2	0.97	2.42	3.069 (2)	124

**Fig. 1.**
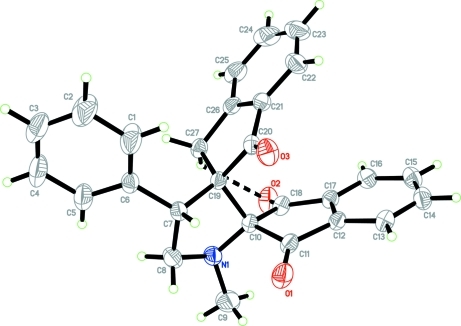
The molecular structure of the title compound, showing 30% probability displacement ellipsoids for non-H atoms. The intramolecular hydrogen bond is shown as a dashed line.

## supplementary crystallographic information

### Comment

Heterocyclic compounds, especially those with five- and six-membered rings have received considerable attention as a result of their diverse biological activities (Amalraj *et al.*, 2003). Substituted pyrrolidine analogues have high potential in the treatment of tuberculosis (TB) (Karthikeyan *et al.*, 2010). Tuberculosis, an illness caused by Mycobacterium tuberculosis, is the leading cause of worldwide death from infectious diseases (Chande *et al.*, 2005). Problems arise when patients develop bacterial resistance to the first-line drugs and the second-line drugs are too toxic and cannot be employed simultaneously (Sriram *et al.*, 2009). Therefore, there is an impetus for the development of new antitubercular agents to shorten the treatment regime and which are effective against drug-resistant strains of M. tuberculosis (Duncan & Barry, 2004).

The molecular structure is shown in Fig. 1. Bond lengths (Allen *et al.*, 1987) and angles are within normal ranges and are comparable to those in related crystal structures (Kumar *et al.*, 2010; Wei *et al.*, 2011).

The pyrrolidine ring (N1/C7/C8/C10/C19) is twisted about the C10—C19 bond, with puckering parameters (Cremer & Pople, 1975) Q = 0.4335 (17) Å and φ = 126.7 (2)°, thereby adopting a half-chair conformation. The two five-membered carbocyclic rings, C10-C12/C17/C18 and C19-C21/C26/C27, are in envelope conformations: puckering parameters Q = 0.1992 (17) Å and φ = 179.8 (5)° with atom C10 at the flap; and Q = 0.1879 (16) Å and φ = 355.8 (5)°  with atom C19 at the flap, respectively.

If the three benzene rings C1–C6, C12–C17 and C21–C26 are denoted by R4, R5, R6 then the dihedral angles for R4^R5, R5^R6 and R4^R6 are 79.88 (11), 34.21 (9) and 82.39 (12)°, respectively.

The molecular structure is stabilized by an intramolecular C27–H27A···O2 hydrogen bond (Table 1), which generates an *S*(6) ring motif (Fig. 1, Bernstein *et al.*, 1995). No significant intermolecular hydrogen bond is observed. There is a short contact of 1.94 Å between H5A and H8B.

### Experimental

A mixture of (*E*)-(2-benzylidene)-2,3-dihydro-IH-indene-1-one (0.001 mmol), ninhydrin (0.001 mmol) and sarcosine (0.002 mmol) (1:1:2) was dissolved in methanol (10 ml) and refluxed for 4 h. After completion of the reaction, as evident from TLC, the mixture was poured into water (50 ml). The precipitated solid was filtered, washed with water and recrystallised from a pet. ether - ethyl acetate mixture (1:1) to yield the title compound as yellow crystals.

### Refinement

All H atoms were positioned geometrically and refined using a riding model with C–H = 0.93-0.98 Å and *U*_iso_(H) = 1.2 or 1.5 *U*_eq_(C). A rotating-group model was applied for the methyl group.

### Crystal data

**Table:**

C_27_H_21_NO_3_	*F*(000) = 856
*M**_r_* = 407.45	*D*_x_ = 1.292 Mg m^−^^3^
Monoclinic, *P*2_1_/*c*	Mo *K*α radiation, λ = 0.71073 Å
Hall symbol: -P 2ybc	Cell parameters from 5963 reflections
*a* = 8.4578 (7) Å	θ = 2.6–28.2°
*b* = 11.6194 (9) Å	µ = 0.08 mm^−^^1^
*c* = 22.6360 (16) Å	*T* = 296 K
β = 109.693 (2)°	Block, yellow
*V* = 2094.4 (3) Å^3^	0.34 × 0.26 × 0.15 mm
*Z* = 4	

### Data collection

**Table:**

Bruker SMART APEXII DUO CCD area-detector diffractometer	6084 independent reflections
Radiation source: fine-focus sealed tube	3982 reflections with *I* > 2σ(*I*)
graphite	*R*_int_ = 0.035
φ and ω scans	θ_max_ = 30.1°, θ_min_ = 1.9°
Absorption correction: multi-scan (*SADABS*; Bruker, 2009)	*h* = −11→11
*T*_min_ = 0.972, *T*_max_ = 0.988	*k* = −15→16
23854 measured reflections	*l* = −31→31

### Refinement

**Table:**

Refinement on *F*^2^	Primary atom site location: structure-invariant direct methods
Least-squares matrix: full	Secondary atom site location: difference Fourier map
*R*[*F*^2^ > 2σ(*F*^2^)] = 0.052	Hydrogen site location: inferred from neighbouring sites
*wR*(*F*^2^) = 0.163	H-atom parameters constrained
*S* = 1.03	*w* = 1/[σ^2^(*F*_o_^2^) + (0.0731*P*)^2^ + 0.4081*P*] where *P* = (*F*_o_^2^ + 2*F*_c_^2^)/3
6084 reflections	(Δ/σ)_max_ = 0.001
281 parameters	Δρ_max_ = 0.20 e Å^−^^3^
0 restraints	Δρ_min_ = −0.21 e Å^−^^3^

### Special details

**Table:**

Geometry. All esds (except the esd in the dihedral angle between two l.s. planes) are estimated using the full covariance matrix. The cell esds are taken into account individually in the estimation of esds in distances, angles and torsion angles; correlations between esds in cell parameters are only used when they are defined by crystal symmetry. An approximate (isotropic) treatment of cell esds is used for estimating esds involving l.s. planes.
Refinement. Refinement of F^2^ against ALL reflections. The weighted R-factor wR and goodness of fit S are based on F^2^, conventional R-factors R are based on F, with F set to zero for negative F^2^. The threshold expression of F^2^ > 2sigma(F^2^) is used only for calculating R-factors(gt) etc. and is not relevant to the choice of reflections for refinement. R-factors based on F^2^ are statistically about twice as large as those based on F, and R- factors based on ALL data will be even larger.

### Fractional atomic coordinates and isotropic or equivalent isotropic displacement parameters (Å^2^)

**Table:**

	*x*	*y*	*z*	*U*_iso_*/*U*_eq_	
O1	1.34827 (16)	0.31094 (11)	0.95641 (6)	0.0717 (4)	
O2	1.00814 (19)	0.39487 (11)	0.75292 (5)	0.0679 (4)	
O3	0.98637 (17)	0.36481 (11)	0.97255 (5)	0.0622 (3)	
N1	1.15973 (17)	0.19994 (11)	0.83324 (7)	0.0517 (3)	
C1	0.7927 (4)	0.0968 (2)	0.94738 (12)	0.0926 (8)	
H1A	0.8054	0.1696	0.9654	0.111*	
C2	0.6731 (4)	0.0227 (2)	0.95567 (14)	0.1032 (9)	
H2A	0.6047	0.0476	0.9778	0.124*	
C3	0.6549 (3)	−0.0845 (2)	0.93209 (12)	0.0863 (7)	
H3A	0.5778	−0.1352	0.9390	0.104*	
C4	0.7512 (4)	−0.1166 (2)	0.89819 (17)	0.1153 (10)	
H4A	0.7393	−0.1902	0.8812	0.138*	
C5	0.8678 (3)	−0.04216 (18)	0.88813 (14)	0.0938 (8)	
H5A	0.9306	−0.0661	0.8637	0.113*	
C6	0.89185 (19)	0.06577 (13)	0.91353 (7)	0.0457 (3)	
C7	1.02348 (18)	0.14830 (12)	0.90680 (7)	0.0415 (3)	
H7A	1.0976	0.1668	0.9494	0.050*	
C8	1.1369 (2)	0.10356 (15)	0.87084 (10)	0.0607 (5)	
H8A	1.2442	0.0783	0.8999	0.073*	
H8B	1.0839	0.0394	0.8441	0.073*	
C9	1.3153 (3)	0.2003 (2)	0.81922 (12)	0.0852 (7)	
H9A	1.3127	0.2618	0.7906	0.128*	
H9B	1.3275	0.1282	0.8005	0.128*	
H9C	1.4084	0.2112	0.8573	0.128*	
C10	1.11262 (17)	0.30515 (12)	0.85686 (7)	0.0414 (3)	
C11	1.24588 (18)	0.36203 (13)	0.91420 (7)	0.0466 (3)	
C12	1.22771 (17)	0.48842 (13)	0.90556 (7)	0.0424 (3)	
C13	1.2992 (2)	0.57672 (14)	0.94769 (8)	0.0504 (4)	
H13A	1.3671	0.5608	0.9886	0.060*	
C14	1.2661 (2)	0.68821 (15)	0.92684 (9)	0.0575 (4)	
H14A	1.3126	0.7485	0.9542	0.069*	
C15	1.1648 (2)	0.71263 (15)	0.86571 (9)	0.0599 (4)	
H15A	1.1453	0.7889	0.8529	0.072*	
C16	1.0922 (2)	0.62575 (14)	0.82347 (8)	0.0523 (4)	
H16A	1.0239	0.6422	0.7826	0.063*	
C17	1.12528 (18)	0.51301 (13)	0.84439 (7)	0.0428 (3)	
C18	1.07040 (19)	0.40499 (14)	0.80921 (7)	0.0461 (3)	
C19	0.95609 (16)	0.26431 (12)	0.87430 (6)	0.0378 (3)	
C20	0.91217 (19)	0.35346 (12)	0.91692 (7)	0.0428 (3)	
C21	0.76765 (19)	0.42024 (13)	0.87730 (8)	0.0477 (4)	
C22	0.7002 (3)	0.52158 (15)	0.89235 (11)	0.0663 (5)	
H22A	0.7461	0.5559	0.9316	0.080*	
C23	0.5639 (3)	0.5683 (2)	0.84708 (15)	0.0856 (7)	
H23A	0.5155	0.6354	0.8555	0.103*	
C24	0.4974 (3)	0.5161 (2)	0.78866 (14)	0.0898 (8)	
H24A	0.4044	0.5491	0.7588	0.108*	
C25	0.5656 (2)	0.41625 (19)	0.77349 (10)	0.0710 (6)	
H25A	0.5206	0.3829	0.7340	0.085*	
C26	0.70320 (18)	0.36772 (13)	0.81919 (8)	0.0477 (4)	
C27	0.79686 (18)	0.25889 (13)	0.81565 (7)	0.0456 (3)	
H27A	0.8256	0.2571	0.7776	0.055*	
H27B	0.7304	0.1914	0.8167	0.055*	

### Atomic displacement parameters (Å^2^)

**Table:**

	*U*^11^	*U*^22^	*U*^33^	*U*^12^	*U*^13^	*U*^23^
O1	0.0569 (7)	0.0572 (7)	0.0736 (8)	−0.0085 (6)	−0.0143 (6)	0.0166 (6)
O2	0.0945 (10)	0.0639 (8)	0.0383 (6)	−0.0176 (7)	0.0131 (6)	0.0000 (5)
O3	0.0805 (8)	0.0580 (7)	0.0440 (7)	−0.0050 (6)	0.0155 (6)	−0.0125 (5)
N1	0.0550 (7)	0.0446 (7)	0.0638 (9)	−0.0008 (6)	0.0309 (7)	−0.0027 (6)
C1	0.128 (2)	0.0670 (13)	0.119 (2)	−0.0339 (13)	0.0878 (18)	−0.0193 (13)
C2	0.122 (2)	0.0954 (19)	0.125 (2)	−0.0344 (16)	0.0859 (18)	−0.0022 (16)
C3	0.0723 (13)	0.0836 (16)	0.0965 (17)	−0.0276 (12)	0.0200 (12)	0.0272 (13)
C4	0.119 (2)	0.0597 (14)	0.181 (3)	−0.0419 (14)	0.069 (2)	−0.0216 (17)
C5	0.0971 (16)	0.0507 (11)	0.155 (2)	−0.0228 (11)	0.0698 (17)	−0.0251 (13)
C6	0.0477 (7)	0.0391 (7)	0.0461 (8)	−0.0035 (6)	0.0103 (6)	0.0053 (6)
C7	0.0449 (7)	0.0352 (7)	0.0431 (8)	−0.0017 (5)	0.0130 (6)	0.0005 (6)
C8	0.0684 (11)	0.0437 (9)	0.0810 (13)	0.0068 (8)	0.0396 (10)	0.0044 (8)
C9	0.0804 (14)	0.0779 (14)	0.122 (2)	0.0045 (11)	0.0670 (14)	0.0036 (13)
C10	0.0420 (7)	0.0378 (7)	0.0428 (8)	−0.0054 (5)	0.0120 (6)	−0.0010 (6)
C11	0.0398 (7)	0.0463 (8)	0.0475 (8)	−0.0071 (6)	0.0064 (6)	0.0056 (7)
C12	0.0391 (6)	0.0417 (7)	0.0457 (8)	−0.0060 (6)	0.0131 (6)	0.0017 (6)
C13	0.0497 (8)	0.0520 (9)	0.0476 (8)	−0.0098 (7)	0.0139 (7)	−0.0029 (7)
C14	0.0677 (10)	0.0469 (9)	0.0613 (11)	−0.0081 (8)	0.0260 (9)	−0.0119 (8)
C15	0.0736 (11)	0.0420 (9)	0.0670 (11)	0.0037 (8)	0.0275 (9)	0.0034 (8)
C16	0.0571 (9)	0.0498 (9)	0.0498 (9)	0.0016 (7)	0.0176 (7)	0.0083 (7)
C17	0.0429 (7)	0.0429 (8)	0.0426 (8)	−0.0046 (6)	0.0146 (6)	0.0033 (6)
C18	0.0485 (8)	0.0489 (8)	0.0396 (8)	−0.0080 (6)	0.0132 (6)	0.0010 (6)
C19	0.0380 (6)	0.0349 (7)	0.0383 (7)	−0.0038 (5)	0.0098 (5)	−0.0032 (5)
C20	0.0477 (7)	0.0362 (7)	0.0453 (8)	−0.0065 (6)	0.0170 (6)	−0.0035 (6)
C21	0.0480 (8)	0.0405 (8)	0.0611 (10)	0.0006 (6)	0.0267 (7)	0.0062 (7)
C22	0.0780 (12)	0.0463 (9)	0.0926 (14)	0.0087 (8)	0.0524 (11)	0.0089 (9)
C23	0.0805 (14)	0.0618 (13)	0.134 (2)	0.0262 (11)	0.0619 (16)	0.0306 (14)
C24	0.0582 (11)	0.0838 (16)	0.129 (2)	0.0260 (11)	0.0339 (13)	0.0571 (16)
C25	0.0481 (9)	0.0771 (13)	0.0796 (13)	0.0005 (9)	0.0108 (9)	0.0307 (11)
C26	0.0370 (7)	0.0469 (8)	0.0578 (9)	−0.0028 (6)	0.0141 (6)	0.0135 (7)
C27	0.0434 (7)	0.0443 (8)	0.0428 (8)	−0.0079 (6)	0.0064 (6)	−0.0026 (6)

### Geometric parameters (Å, °)

**Table:**

O1—C11	1.2073 (18)	C11—C12	1.483 (2)
O2—C18	1.2092 (18)	C12—C13	1.393 (2)
O3—C20	1.2102 (18)	C12—C17	1.393 (2)
N1—C10	1.4432 (19)	C13—C14	1.375 (2)
N1—C9	1.454 (2)	C13—H13A	0.9300
N1—C8	1.459 (2)	C14—C15	1.389 (3)
C1—C6	1.362 (3)	C14—H14A	0.9300
C1—C2	1.388 (3)	C15—C16	1.384 (2)
C1—H1A	0.9300	C15—H15A	0.9300
C2—C3	1.344 (4)	C16—C17	1.389 (2)
C2—H2A	0.9300	C16—H16A	0.9300
C3—C4	1.346 (4)	C17—C18	1.475 (2)
C3—H3A	0.9300	C19—C27	1.5411 (19)
C4—C5	1.387 (3)	C19—C20	1.5438 (19)
C4—H4A	0.9300	C20—C21	1.470 (2)
C5—C6	1.366 (3)	C21—C26	1.384 (2)
C5—H5A	0.9300	C21—C22	1.400 (2)
C6—C7	1.515 (2)	C22—C23	1.370 (3)
C7—C8	1.542 (2)	C22—H22A	0.9300
C7—C19	1.549 (2)	C23—C24	1.390 (4)
C7—H7A	0.9800	C23—H23A	0.9300
C8—H8A	0.9700	C24—C25	1.390 (3)
C8—H8B	0.9700	C24—H24A	0.9300
C9—H9A	0.9600	C25—C26	1.389 (2)
C9—H9B	0.9600	C25—H25A	0.9300
C9—H9C	0.9600	C26—C27	1.508 (2)
C10—C18	1.542 (2)	C27—H27A	0.9700
C10—C11	1.551 (2)	C27—H27B	0.9700
C10—C19	1.5766 (19)		
			
C10—N1—C9	117.49 (14)	C14—C13—C12	117.91 (16)
C10—N1—C8	109.06 (12)	C14—C13—H13A	121.0
C9—N1—C8	115.95 (15)	C12—C13—H13A	121.0
C6—C1—C2	121.8 (2)	C13—C14—C15	121.34 (16)
C6—C1—H1A	119.1	C13—C14—H14A	119.3
C2—C1—H1A	119.1	C15—C14—H14A	119.3
C3—C2—C1	120.8 (2)	C16—C15—C14	121.36 (16)
C3—C2—H2A	119.6	C16—C15—H15A	119.3
C1—C2—H2A	119.6	C14—C15—H15A	119.3
C2—C3—C4	118.2 (2)	C15—C16—C17	117.45 (15)
C2—C3—H3A	120.9	C15—C16—H16A	121.3
C4—C3—H3A	120.9	C17—C16—H16A	121.3
C3—C4—C5	121.4 (2)	C16—C17—C12	121.24 (14)
C3—C4—H4A	119.3	C16—C17—C18	128.89 (14)
C5—C4—H4A	119.3	C12—C17—C18	109.83 (13)
C6—C5—C4	121.1 (2)	O2—C18—C17	126.70 (14)
C6—C5—H5A	119.4	O2—C18—C10	125.44 (14)
C4—C5—H5A	119.4	C17—C18—C10	107.82 (12)
C1—C6—C5	116.57 (17)	C27—C19—C20	103.96 (11)
C1—C6—C7	120.11 (15)	C27—C19—C7	116.64 (11)
C5—C6—C7	123.30 (16)	C20—C19—C7	114.48 (12)
C6—C7—C8	116.63 (13)	C27—C19—C10	111.13 (11)
C6—C7—C19	115.67 (12)	C20—C19—C10	110.71 (11)
C8—C7—C19	103.73 (12)	C7—C19—C10	100.09 (11)
C6—C7—H7A	106.7	O3—C20—C21	127.61 (14)
C8—C7—H7A	106.7	O3—C20—C19	125.02 (14)
C19—C7—H7A	106.7	C21—C20—C19	107.37 (12)
N1—C8—C7	106.11 (12)	C26—C21—C22	122.67 (16)
N1—C8—H8A	110.5	C26—C21—C20	109.10 (13)
C7—C8—H8A	110.5	C22—C21—C20	128.23 (17)
N1—C8—H8B	110.5	C23—C22—C21	117.4 (2)
C7—C8—H8B	110.5	C23—C22—H22A	121.3
H8A—C8—H8B	108.7	C21—C22—H22A	121.3
N1—C9—H9A	109.5	C22—C23—C24	120.5 (2)
N1—C9—H9B	109.5	C22—C23—H23A	119.7
H9A—C9—H9B	109.5	C24—C23—H23A	119.7
N1—C9—H9C	109.5	C23—C24—C25	122.1 (2)
H9A—C9—H9C	109.5	C23—C24—H24A	119.0
H9B—C9—H9C	109.5	C25—C24—H24A	119.0
N1—C10—C18	113.94 (12)	C26—C25—C24	117.9 (2)
N1—C10—C11	117.22 (13)	C26—C25—H25A	121.0
C18—C10—C11	101.33 (11)	C24—C25—H25A	121.0
N1—C10—C19	101.41 (11)	C21—C26—C25	119.43 (17)
C18—C10—C19	112.68 (12)	C21—C26—C27	111.98 (13)
C11—C10—C19	110.70 (11)	C25—C26—C27	128.58 (17)
O1—C11—C12	127.32 (14)	C26—C27—C19	104.01 (12)
O1—C11—C10	125.30 (15)	C26—C27—H27A	111.0
C12—C11—C10	107.33 (12)	C19—C27—H27A	111.0
C13—C12—C17	120.70 (14)	C26—C27—H27B	111.0
C13—C12—C11	129.62 (14)	C19—C27—H27B	111.0
C17—C12—C11	109.66 (13)	H27A—C27—H27B	109.0
			
C6—C1—C2—C3	2.3 (5)	N1—C10—C18—O2	32.0 (2)
C1—C2—C3—C4	−2.5 (5)	C11—C10—C18—O2	158.81 (16)
C2—C3—C4—C5	0.7 (5)	C19—C10—C18—O2	−82.9 (2)
C3—C4—C5—C6	1.5 (5)	N1—C10—C18—C17	−145.92 (13)
C2—C1—C6—C5	0.0 (4)	C11—C10—C18—C17	−19.09 (15)
C2—C1—C6—C7	−178.7 (2)	C19—C10—C18—C17	99.24 (14)
C4—C5—C6—C1	−1.8 (4)	C6—C7—C19—C27	−42.73 (18)
C4—C5—C6—C7	176.8 (2)	C8—C7—C19—C27	86.27 (15)
C1—C6—C7—C8	176.65 (19)	C6—C7—C19—C20	78.95 (15)
C5—C6—C7—C8	−1.9 (3)	C8—C7—C19—C20	−152.06 (13)
C1—C6—C7—C19	−61.0 (2)	C6—C7—C19—C10	−162.65 (12)
C5—C6—C7—C19	120.5 (2)	C8—C7—C19—C10	−33.65 (14)
C10—N1—C8—C7	16.59 (18)	N1—C10—C19—C27	−80.25 (14)
C9—N1—C8—C7	151.92 (17)	C18—C10—C19—C27	41.96 (16)
C6—C7—C8—N1	140.91 (14)	C11—C10—C19—C27	154.64 (12)
C19—C7—C8—N1	12.50 (17)	N1—C10—C19—C20	164.75 (12)
C9—N1—C10—C18	66.1 (2)	C18—C10—C19—C20	−73.04 (15)
C8—N1—C10—C18	−159.39 (14)	C11—C10—C19—C20	39.63 (16)
C9—N1—C10—C11	−52.0 (2)	N1—C10—C19—C7	43.60 (13)
C8—N1—C10—C11	82.57 (16)	C18—C10—C19—C7	165.81 (12)
C9—N1—C10—C19	−172.61 (16)	C11—C10—C19—C7	−81.52 (13)
C8—N1—C10—C19	−38.06 (16)	C27—C19—C20—O3	162.64 (14)
N1—C10—C11—O1	−34.0 (2)	C7—C19—C20—O3	34.26 (19)
C18—C10—C11—O1	−158.66 (17)	C10—C19—C20—O3	−77.95 (18)
C19—C10—C11—O1	81.6 (2)	C27—C19—C20—C21	−17.88 (14)
N1—C10—C11—C12	143.58 (13)	C7—C19—C20—C21	−146.26 (12)
C18—C10—C11—C12	18.94 (15)	C10—C19—C20—C21	101.54 (13)
C19—C10—C11—C12	−100.80 (14)	O3—C20—C21—C26	−169.99 (15)
O1—C11—C12—C13	−13.2 (3)	C19—C20—C21—C26	10.55 (16)
C10—C11—C12—C13	169.23 (15)	O3—C20—C21—C22	10.7 (3)
O1—C11—C12—C17	165.01 (17)	C19—C20—C21—C22	−168.81 (15)
C10—C11—C12—C17	−12.52 (16)	C26—C21—C22—C23	0.6 (2)
C17—C12—C13—C14	−0.4 (2)	C20—C21—C22—C23	179.92 (17)
C11—C12—C13—C14	177.69 (16)	C21—C22—C23—C24	−0.3 (3)
C12—C13—C14—C15	0.0 (2)	C22—C23—C24—C25	−0.5 (3)
C13—C14—C15—C16	0.4 (3)	C23—C24—C25—C26	0.9 (3)
C14—C15—C16—C17	−0.3 (3)	C22—C21—C26—C25	−0.2 (2)
C15—C16—C17—C12	0.0 (2)	C20—C21—C26—C25	−179.63 (14)
C15—C16—C17—C18	−177.50 (16)	C22—C21—C26—C27	−179.01 (14)
C13—C12—C17—C16	0.4 (2)	C20—C21—C26—C27	1.59 (17)
C11—C12—C17—C16	−178.01 (14)	C24—C25—C26—C21	−0.5 (3)
C13—C12—C17—C18	178.31 (13)	C24—C25—C26—C27	178.03 (16)
C11—C12—C17—C18	−0.12 (17)	C21—C26—C27—C19	−12.89 (16)
C16—C17—C18—O2	12.6 (3)	C25—C26—C27—C19	168.47 (16)
C12—C17—C18—O2	−165.04 (16)	C20—C19—C27—C26	18.05 (14)
C16—C17—C18—C10	−169.50 (15)	C7—C19—C27—C26	145.09 (12)
C12—C17—C18—C10	12.82 (16)	C10—C19—C27—C26	−101.08 (13)

### Hydrogen-bond geometry (Å, °)

**Table:**

*D*—H···*A*	*D*—H	H···*A*	*D*···*A*	*D*—H···*A*
C27—H27A···O2	0.97	2.42	3.069 (2)	124
